# Ceftriaxone-Induced Neutropenia Successfully Treated With Alternative β-Lactam Antibiotics: A Case Report and Review of the Literature

**DOI:** 10.7759/cureus.39176

**Published:** 2023-05-18

**Authors:** Kana Satake, Kenta Iijima

**Affiliations:** 1 Department of Internal Medicine, Hyogo Prefectural Amagasaki General Medical Center: Hyogo Kenritsu Amagasaki Sogo Iryo Center, Amagasaki, JPN; 2 Department of Infectious Diseases, Hyogo Prefectural Amagasaki General Medical Center: Hyogo Kenritsu Amagasaki Sogo Iryo Center, Amagasaki, JPN

**Keywords:** case report, cross-reactivity, infective endocarditis, ampicillin sodium, neutropenia, ceftriaxone

## Abstract

Ceftriaxone-induced neutropenia is a rare and severe adverse effect of the drug. It usually resolves in one to three weeks following the cessation of ceftriaxone and the administration of granulocyte colony-stimulating factor (G-CSF). After neutrophil recovery, patients are often treated with non-β-lactam antibiotics instead of ceftriaxone due to the possibility of cross-reactivity associated with β-lactam allergy. However, in some cases, β-lactam antibiotics are superior to non-β-lactam antibiotics. Few cases of the readministration of β-lactam antibiotics for patients who developed ceftriaxone-induced neutropenia have been reported so far. Moreover, its pathogenesis and management have still not been established. We describe a case of successful readministration of β-lactam antibiotics for a patient who had developed ceftriaxone-induced neutropenia. A 37-year-old man with a prosthetic aortic valve was admitted to our hospital with a fever. Blood culture on admission revealed methicillin-susceptible Staphylococcus aureus (MSSA) bacteremia, and transesophageal echocardiography (TEE) showed aortic valve vegetation with multiple septic emboli seen on brain CT. We diagnosed MSSA infective endocarditis with central nervous complications. He underwent an operation and was treated with ceftriaxone. On admission day 28, he developed neutropenia (33/μL), and ceftriaxone-induced neutropenia was suspected. Vancomycin was started instead of ceftriaxone, and his neutrophil count recovered within two weeks with the administration of G-CSF. After recovery, on day 40 of admission, ampicillin sodium was administered instead of vancomycin. Although he developed mild eosinophilia, he did not exhibit neutropenia and was discharged with an amoxicillin prescription on day 60 of admission.

Our report suggests the possibility that patients who develop ceftriaxone-induced neutropenia can be treated safely with an alternative β-lactam antibiotic, ampicillin sodium, without causing β-lactam cross-reactivity of neutropenia.

## Introduction

Drug-induced neutropenia is a rare and severe adverse reaction characterized by a decrease in the peripheral neutrophil count to less than 0.5 × 10^9^ cells/L; the condition is considered life-threatening and associated with a high mortality rate (~5%) [1]. Ceftriaxone, a third-generation cephalosporin antibiotic used to treat various infectious diseases, is reported to cause some adverse reactions, and most cases are related to skin and appendage disorders [2]. Ceftriaxone-induced neutropenia has also been reported in a few clinical cases. Despite β-lactam antibiotics being superior to non-β-lactam antibiotics in certain situations, such as for treating methicillin-susceptible Staphylococcus aureus (MSSA) bacteremia [3], few studies have examined the use of alternative β-lactam antibiotics for the treatment of ceftriaxone-induced neutropenia [4,5,6]. We describe a case of a male patient who developed neutropenia while receiving high doses of ceftriaxone (4 g/day) for infective endocarditis with central nervous complications, which resolved after the cessation of the medication; his neutropenia did not relapse after the medication was replaced with ampicillin sodium. We also review previously reported cases of β-lactam-induced neutropenia and discuss the possible options for its management.

## Case presentation

**Figure 1:**
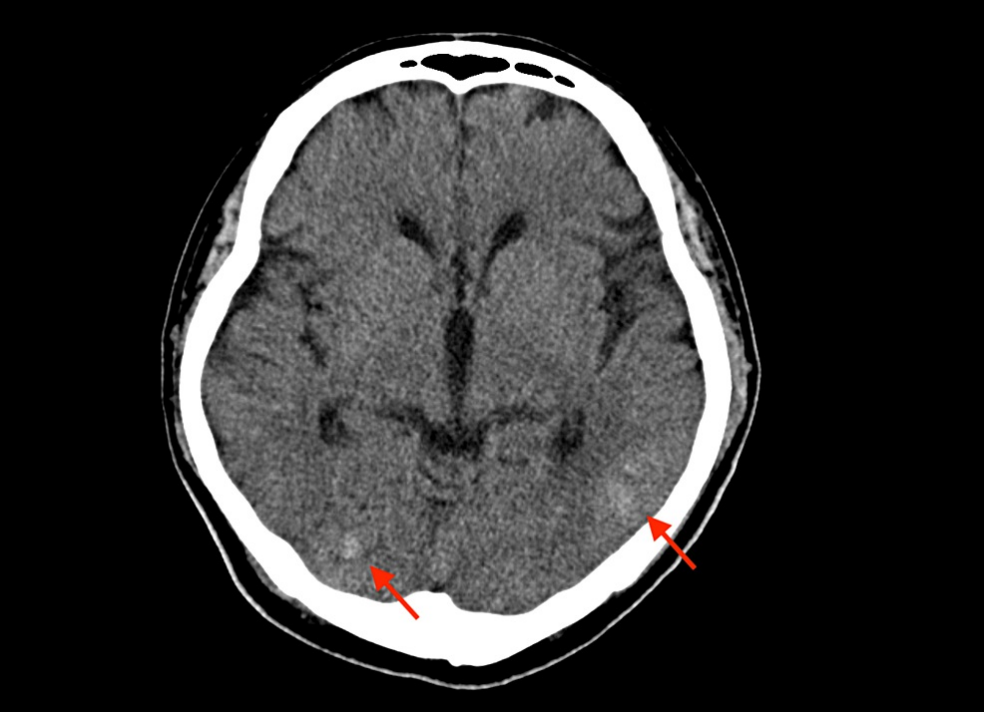
Brain CT showing multiple septic emboli involving bilateral cerebrum (arrows) CT: computed tomography

**Figure 2:**
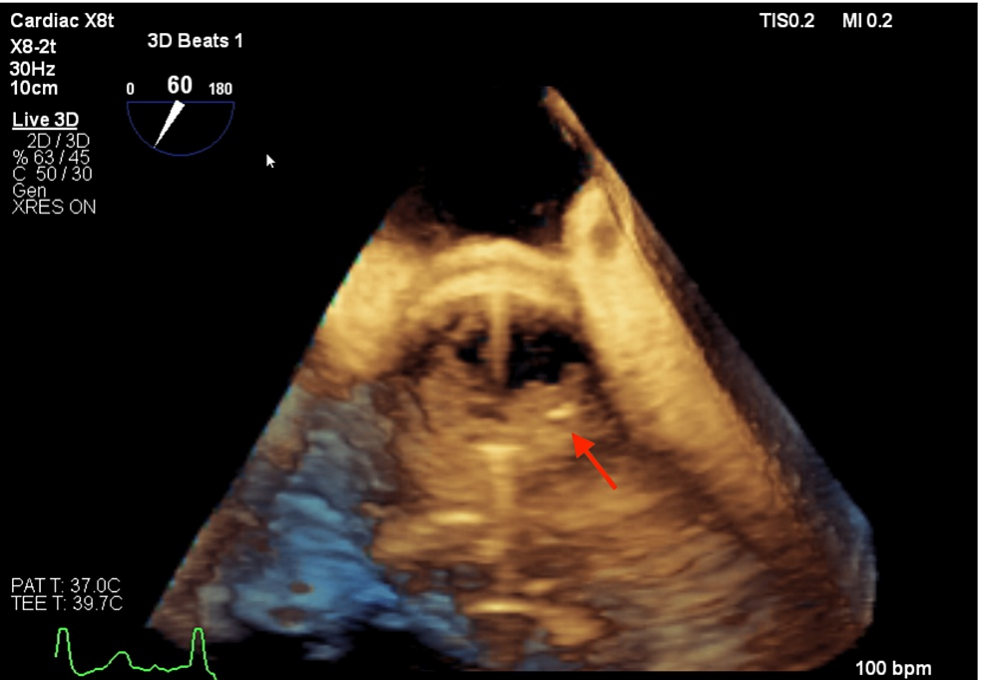
TEE showing perivalvular abscess on an aortic prosthetic valve (arrow) TEE: transesophageal echocardiography

A 37-year-old male patient with a previous diagnosis of aortic root enlargement who had undergone aortic root replacement was admitted to the emergency department with a fever. There was no specific medical history other than the aortic root enlargement. His body temperature was 40 °C, pulse was 132/minute, and systolic blood pressure was about 170 mmHg. The patient's blood culture was positive for MSSA. Brain CT revealed several septic emboli and transesophageal echocardiography (TEE) detected a perivalvular abscess on an aortic prosthetic valve (Figures 1, 2).

We diagnosed prosthetic aortic valve infective endocarditis with central nervous system complications. On admission, the patient was treated with empiric therapy using a high dose of ceftriaxone (4 g/day) due to his central nervous system complications. Moreover, the European Committee for Antimicrobial Susceptibility Testing (EUCAST) breakpoint criteria for MSSA treatment with ceftriaxone are based on a high dose (4 g/day) rather than the standard dose (2 g/day) [7]. On day seven of admission, he underwent surgical valve replacement surgery and continued with ceftriaxone. We added aminoglycoside from day eight to day 21 and rifampicin from day 15 to day 27.

On day 23 of admission, he reported nonpruritic facial rashes while receiving ceftriaxone. On day 25, a maculopapular rash appeared on his abdomen and upper limb. His laboratory data showed a mild decline in neutrophil count, hemoglobin levels, and eosinophil count (approximately 500/μL). The platelet, lymphocyte, and erythrocyte counts were unremarkable. We suspected ceftriaxone-induced neutropenia, and ceftriaxone was discontinued and replaced with vancomycin. On day 28, the neutrophil count declined to 33 cells/L, and a bone marrow aspirate was examined. It revealed myelocyte maturational arrest and the growth of erythrocytes, indicating inflammation. Over the following week, the number of neutrophils completely recovered upon cessation of ceftriaxone and granulocyte colony-stimulating factor (G-CSF) administration. We administered filgrastim 75 μg/day from day 28 to day 31. On day 40 of admission, vancomycin was discontinued and replaced with ampicillin sodium as vancomycin is inferior to β-lactam in treating MSSA bacteremia. After his eosinophil counts increased by two weeks after the initiation of ampicillin sodium and reached a high of 750/μL, the counts stagnated for the remaining period without the development of neutropenia. On day 60 of admission, he was discharged with an oral amoxicillin prescription.

**Figure 3:**
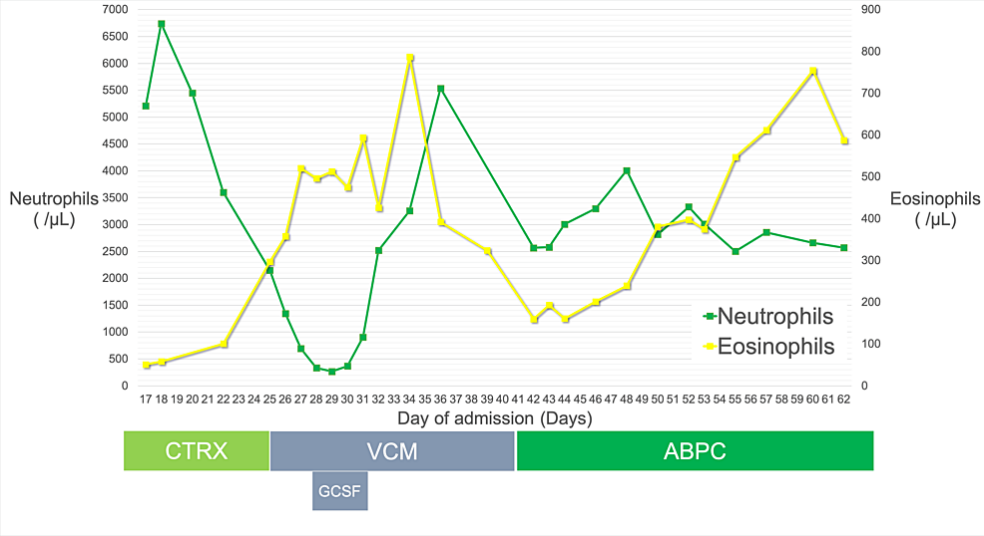
Clinical course and neutrophil counts CTRX: ceftriaxone; VCM: vancomycin; ABPC: amoxicillin sodium; G-CSF: granulocyte colony-stimulating factor

Figure 3 depicts the clinical course and neutrophil counts of the patient.

## Discussion

The incidence of nonchemotherapy idiosyncratic drug-induced neutropenia is reported to be 2.4-15.4 cases per million people [8]. However, ceftriaxone-induced neutropenia is rare. Andersohn et al. performed a systematic review of case reports from 1966 to 2006 involving nonchemotherapy drug-induced neutropenia, and they found that only six cases out of 980 were caused by ceftriaxone [9]. In our case, the Naranjo criteria score, based on 10 items and the estimated probabilities of the culprit drug, was 7, indicating a possible ceftriaxone-induced adverse drug reaction [10]. There are various reports about the cumulative dose or treatment duration. Neftel et al. analyzed the relationship between the dose and the treatment duration in 50 of their patients who exhibited β-lactam-induced neutropenia. Six cases were induced by ceftriaxone; the mean treatment duration was 21 days (8-25 days), and the mean dose was 51 ±29 g [11]. Furthermore, the coincidence of eosinophilia or rash may occur [12]. Our patient developed neutropenia after a 28-day ceftriaxone therapy with a cumulative dose of 112 g.

Management of drug-induced neutropenia includes the immediate discontinuation of the offending medication and awaiting the recovery of the neutrophil count [13,14,15]. Neutrophils usually recover within one to three weeks after the cessation of the culprit drug, and treatment with G-CSF administration is associated with shorter recovery times [13,14,15].

While the pathogenesis of ceftriaxone-induced neutropenia remains to be elucidated, it is thought to be due to immunologic or cytotoxic mechanisms [16]. Some investigations have mentioned clinically important immunologic cross-reactivity in patients with confirmed cephalosporin allergy to penicillin. Therefore, the potential risk cannot be underestimated. Hence, other β-lactam agents are rarely readministered to patients who have previously exhibited β-lactam allergies. Patients with ceftriaxone-induced neutropenia are often treated with alternative non-β-lactam antibiotics.

We searched PubMed and Google Scholar in Japanese from inception to April 2023, and each search query included the terms "(Ceftriaxone) AND ((neutropenia) OR (agranulocytosis))." Only three reports have described the use of alternative β-lactam antibiotics for patients who developed ceftriaxone-induced neutropenia (Table 1).

**Table 1:** Cases of readministration of other β-lactam antibiotics for patients who developed ceftriaxone-induced neutropenia *1 g every 12 hours for four days and 2 g every 12 hours for the remaining 21 days CFPM: cefepime; MEPM: meropenem; VCM: vancomycin; AMPC: amoxicillin; p.o.: per os; G-CSF: granulocyte colony-stimulating factor

Year	Authors	Age/sex	Microbiology	Indication	Dose per day	Treatment duration	Alternative medicine	G-CSF	Relapse of neutropenia
2015	Uy et al. [4]	49/M	Streptococcus intermedius	Vertebral osteomyelitis	4 g*	25 days	CFPM	No	No
2021	Couto et al. [6]	70/F	Streptococcus pneumoniae	Brain abscess	4 g	29 days	MEPM	Yes	No
2022	Munir et al. [5]	78/M	Streptococcus mitis	Bacteremia of unknown origin	2 g	38 days	VCM + MEPM followed by AMPC p.o.	Yes	No
	Our case	37/M	Staphylococcus aureus	Infective endocarditis	4 g	28 days	VCM followed by ABPC	Yes	No

In cephalosporines, a 6-membered dihydro thiazine ring (cefepime ring) is attached. The R1 chain is attached to the β-lactam ring, and the R2 chain is attached to the cefepime ring [17]. Couto et al. reported that the alternative administration of cefepime with identical R1 chains for a patient with ceftriaxone-induced neutropenia did not cause allergic reactions, including neutropenia, suggesting that neutropenia is assumed to originate from the R2 chain if the pathogenesis involves immunologic reactions [6]. Penicillin and cephalosporines have different R1 chains and share only a common 4-membered β-lactam ring.

In our case, ampicillin sodium was administered after the recovery of the neutrophil count because β-lactam antibiotics are superior to vancomycin as definitive therapy for a patient with MSSA bloodstream infections [5] and because of the unavailability of nafcillin or oxacillin in Japan [18]. Although mild eosinophilia was observed, the patient was also treated safely with ampicillin sodium, which did not cause neutropenia from β-lactam cross-reactivity. Based on this case, we assume that the development of neutropenia and eosinophilia involve distinct mechanisms, with the former originating from the R2 chain and the latter from the β-lactam ring or R1 chain.

## Conclusions

This case report suggests the possibility of the safe initiation of alternative β-lactam antibiotics with different R1 chains or R2 chains for patients who develop ceftriaxone-induced neutropenia. We believe it is not always necessary to completely avoid the readministration of β-lactams because of neutropenia, and even if eosinophilia is observed, it may be possible to use them in some cases. However, the indication for the safe selection of other β-lactam antibiotics has not been conclusively established, and further studies are needed to understand the detailed immunologic mechanisms and to determine which patient groups can safely be treated with these drugs.
